# Microbiome-derived metabolites alleviate chronic pain in a reserpine-induced model of fibromyalgia

**DOI:** 10.1016/j.isci.2026.115406

**Published:** 2026-03-18

**Authors:** Shen Chen, Dhanya Shanmuganathan, Wendy L. Imlach

**Affiliations:** 1 Department of Physiology, Monash Biomedicine Discovery Institute, Monash University, Melbourne, VIC 3800, Australia

**Keywords:** biological sciences, neuroscience, microbiome

## Abstract

Fibromyalgia is a chronic pain disorder driven by central sensitization and neuroinflammation, increasingly linked to gut-brain axis dysfunction. Here, we delineate a gut-to-CNS axis for pain modulation, demonstrating that an acetate-producing diet alleviates reserpine-induced-fibromyalgia in a rodent model. We show that diet rich in acetylated high-amylose maize starch shifts the gut microbiome to favor acetate-producing bacteria, increasing systemic acetate levels and reducing pain hypersensitivity. This is associated with reduced spinal microglia activation and anti-inflammatory cytokine gene expression, with elevated IL-10 mRNA in the DRG and IL-10, IL-2, and IL-6 in the spinal cord. Electrophysiologically, we observe reduced hyperexcitability in the dorsal horn and increased inhibitory activity. The mechanism driving this change involves reduced prostaglandin-E2 (PGE2)-mediated suppression of glycinergic inhibition, a direct consequence of maintaining microglia in quiescent state. These findings link dietary metabolites to reduced fibromyalgia-like pathology and identify targeted nutrition as a potential disease-modifying therapy for chronic pain.

## Highlights

• A high-amylose starch diet reduces fibromyalgia pain through the gut-spinal axis
• In a rat model, this diet reduces microglia activation and neuronal excitability
• Analgesia involves reduced PGE2-suppression of inhibitory neurotransmission

## Graphical abstract

**Figure:**
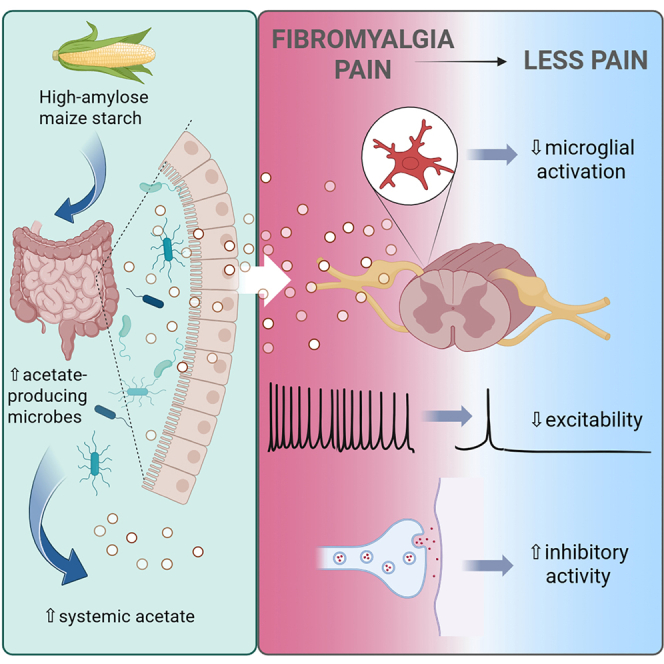

## Introduction

Fibromyalgia is a prevalent and debilitating chronic pain disorder characterized by widespread musculoskeletal pain, fatigue, and cognitive dysfunction, imposing a substantial burden on individuals and healthcare systems.^1^ The pathophysiology of fibromyalgia is complex, but a central unifying mechanism is thought to be due to central sensitization, an amplification of neural signaling within the central nervous system (CNS) that results in pain hypersensitivity.^2^^,^^3^ This state of neuronal hyperexcitability is increasingly understood to be driven and maintained by persistent, low-grade neuroinflammation within pain-processing centers.^4^ This neuroinflammatory involves the activation of glial cells, particularly microglia, which release pro-inflammatory cytokines that alter synaptic transmission,^4^^,^^5^^,^^6^ with clinical studies reporting increases in IL-1β, IL-6, and IL-8 and a decrease in the anti-inflammatory cytokine IL-10.^7^^,^^8^^,^^9^ While much focus has been on the CNS, emerging evidence also implicates peripheral mechanisms, with the dorsal root ganglia (DRG) acting as a critical site where inflammatory mediators can initiate and sustain peripheral sensitization, thereby providing continuous nociceptive drive to the spinal cord.^10^

It has been suggested that the gut-brain axis plays a pivotal role in the modulation of pain and inflammation through interconnected networks that influence host health and disease.^11^ The gut microbiota, through the fermentation of dietary fibers, produces a range of metabolites, including short-chain fatty acids (SCFAs) such as acetate, propionate, and butyrate. These metabolites can enter systemic circulation and exert immunomodulatory and neuro-regulatory effects, both peripherally and within the CNS.^12^^,^^13^ Dysbiosis of the gut microbiota is a recognized feature of fibromyalgia. Clinical studies have revealed a distinct microbial signature in patients, characterized by an enrichment of species like *Flavonifractor plautii* and a depletion of others such as *Faecalibacterium prausnitzii*.^14^ Intriguingly, this dysbiosis is associated with complex metabolic changes, including significantly higher serum levels of butyric acid in patients with fibromyalgia.^14^ The crucial role of the microbiome in these patients was firmly established in a preclinical study showing that transplanting microbiota from fibromyalgia patients is sufficient to induce pain-like behaviors in recipient animals,^15^ positioning the gut microbiome as a promising, yet mechanistically undefined, therapeutic target.

Despite the recognition that neuroinflammation and microbial dysbiosis are key features of fibromyalgia, a direct mechanistic link between a specific gut-derived metabolite and disease pathology remains to be established.^15^ It has been reported that SCFAs can regulate neuroinflammation in other types of chronic pain, including neuropathic pain,^16^^,^^17^ chemotherapy-induced pain,^18^ and complex reginal pain syndrome.^19^ In studies using chronic sciatic nerve constriction models of neuropathic pain SCFAs have been shown to prevent microglial activation and the release of pro-inflammatory cytokines in the hippocampus and spinal cord.^16^ While other studies in rodent neuropathic pain models, have reported effects on macrophage polarization and histone deacetylase (HDAC) expression in the DRG.^17^ However, the mechanism of how these metabolites mediate their analgesic effects across the entire gut-DRG-CNS axis has not been determined. This knowledge gap is particularly critical given that the complex and often contradictory clinical data on SCFA profiles in patients with fibromyalgia, demonstrating the need for detailed mechanistic studies to identify viable therapeutic targets.^14^

Here, we describe a gut-to-spinal cord pathway for pain modulation in a reserpine-induced fibromyalgia model in rats. We show that a targeted dietary intervention designed to enrich acetate-producing bacteria successfully remodels the gut microbiome and alleviates both mechanical and cold allodynia. These changes in the gut microbiome result in a significant elevation of systemic acetate levels and upregulation of IL-10 transcripts in peripheral pain pathways. Centrally, we observe a decrease in spinal microglial activation, which is associated with a shift in the neuro-immune environment toward an anti-inflammatory state, characterized by the increased mRNA expression of IL-10 and the regulatory cytokines IL-2 and IL-6. At the functional level, neuronal hyperexcitability is reduced in the spinal pain pathways, and synaptic balance is restored an attenuation of excitatory drive and restoration of inhibitory activity. Finally, we show these diet-induced changes prevent prostaglandin E2 (PGE2)-mediated suppression of glycinergic inhibitory signaling. Our findings provide a mechanistic framework connecting a specific dietary metabolite to the reversal of fibromyalgia-like pathology and identify a targeted nutritional strategy for the treatment of chronic pain.

## Results

### An acetate-producing diet attenuates mechanical allodynia in a rat model of fibromyalgia-like pain

**Figure 1:**
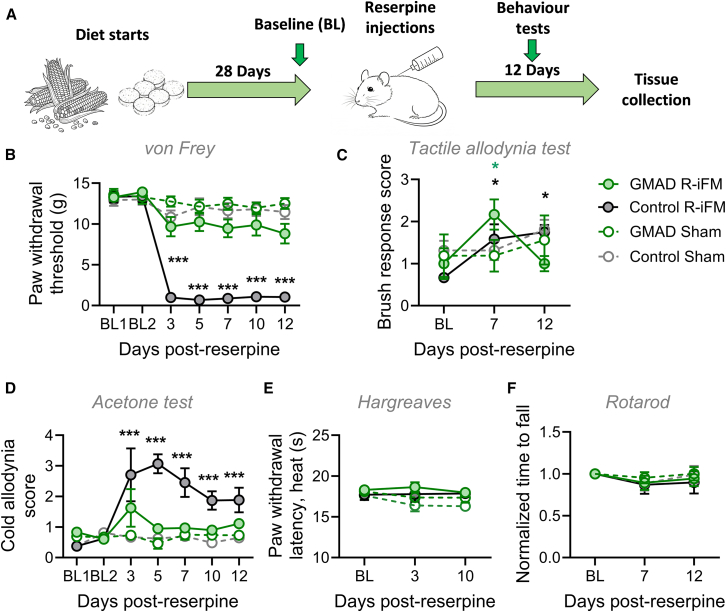
GMAD attenuates reserpine-induced fibromyalgia symptoms (A) Schematic of the experimental timeline. Sprague-Dawley rats were fed GMAD or control diet for 28 days prior to reserpine injection (day 0) to induce R-iFM. Behavioral tests were conducted over a 12-day period following injections and tissues collected on day 13. (B) Mechanical allodynia measured by von Frey (*n* = 12 rats/group, male and female, ∗∗∗*p* < 0.001 compared to sham group). (C) Dynamic allodynia score in response to brush stimuli (*n* = 8 rats/group, ∗*p* < 0.05, compared to baseline). (D) Cold allodynia in response to plantar acetone (*n* = 4–8 rats/group, ∗∗∗*p* < 0.001 compared to sham). (E) Thermal hypersensitivity (*n* = 12 rats/group). (F) Rotarod test normalized to baseline for each group. Data are presented as mean ± SEM. Statistical significance was determined by a two-way repeated ANOVA with Turkey’s post hoc test.

To investigate the therapeutic potential of acetylated high-amylose maize starch in reducing fibromyalgia-like symptoms, we fed male and female adult Sprague-Dawley rats either a control diet, or gut-microbiota acetogenic diet (GMAD) for 28 days, before inducing fibromyalgia-like symptoms through injections of reserpine (0.8 mg/kg) over 3 consecutive days (Figure 1A). Hypersensitivity to mechanical and thermal stimuli was assessed over the 12-day period following reserpine administration. Baseline sensitivity to mechanical stimuli, measured though paw withdrawal threshold (PWT) to von Frey filaments, were not significantly different at baseline across all experimental groups. Following reserpine injections, rats fed the control diet developed sustained mechanical allodynia, with a significant decrease in PWT (13.76 ± 0.71 g to 0.96 ± 0.23 g over 3 days, *p* < 0.001; Figure 1B), which remained low through to day 12. In contrast, the reserpine-induced fibromyalgia (R-iFM) group fed GMAD showed no significant difference in mechanical (13.49 ± 0.58 at baseline to 12.80 ± 0.67 g at day 3, *p* = 0.48; Figure 1B). No significant differences in mechanical allodynia were found between GMAD and control diet groups that were not administered reserpine (sham injection) throughout the experiment (Figure 1B).

To investigate the effect of GMAD on tactile allodynia, the plantar brush test was used at 7- and 12-day post-reserpine injection (Figure 1C). No significant difference from baseline was seen in sham control groups, but significant increases in tactile sensitivity were measured in reserpine treated groups on both GMAD (116.6 ± 35.7% increase from baseline, *p* = 0.039) and control diet (137.5 ± 23.4% increase, *p* = 0.043) at day 7. This increase completely resolved by day 12 for the GMAD R-iFM group, but was still elevated in the control diet R-iFM group (162.5% increase from baseline, *p* = 0.019).

### An acetate-producing diet reduces cold allodynia in a rat model of fibromyalgia-like pain

We next evaluated the effect of GMAD on the development of cold hypersensitivity by applying acetone to the plantar surface of the hind paw (Figure 1D). Similar to the effects on mechanical sensitivity, reserpine-administered rats fed control diet had increased sensitivity to cold, with nocifensive response scores in this group increasing from 0.50 ± 0.09 at baseline to 2.71 ± 0.87 at day 3 (*p* < 0.001), remaining elevated throughout the testing period. GMAD provided significant protection against cold-evoked hypersensitivity with no significant difference in response scores at baseline (0.72 ± 0.09) compared to day 3 (1.63 ± 0.62, *p* = 0.94; Figure 1D). This group displayed significantly lower response scores compared to the control diet R-iFM group at all post-injection time points (days 3, 5, 7, 10, and 12, *p* > 0.05). Interestingly, R-iFM only elevated cold allodynia in the female group, while no significant differences can be seen in male groups (Figure S2).

The Hargreaves test was used to assess hypersensitivity to heat; however, no reserpine-induced changes were observed. There were also no differences in sensitivity seen in the rats fed GMAD compared to the control diet (*p* > 0.05; Figure 1E). To rule out potential confounding effects of motor impairment on behavioral assays, we assessed motor coordination using a rotarod test. No significant differences in mobility activities in rotarod test were observed between any of the experimental groups (*p* > 0.05; Figure 1F), confirming that the observed reductions in pain-like behaviors were not attributable to deficits in motor function. Together, these data establish that the GMAD produces a targeted amelioration of mechanical, tactile, and cold allodynia, key symptoms of fibromyalgia.

### GMAD increases systemic acetate and reshapes the gut microbial community

**Figure 2:**
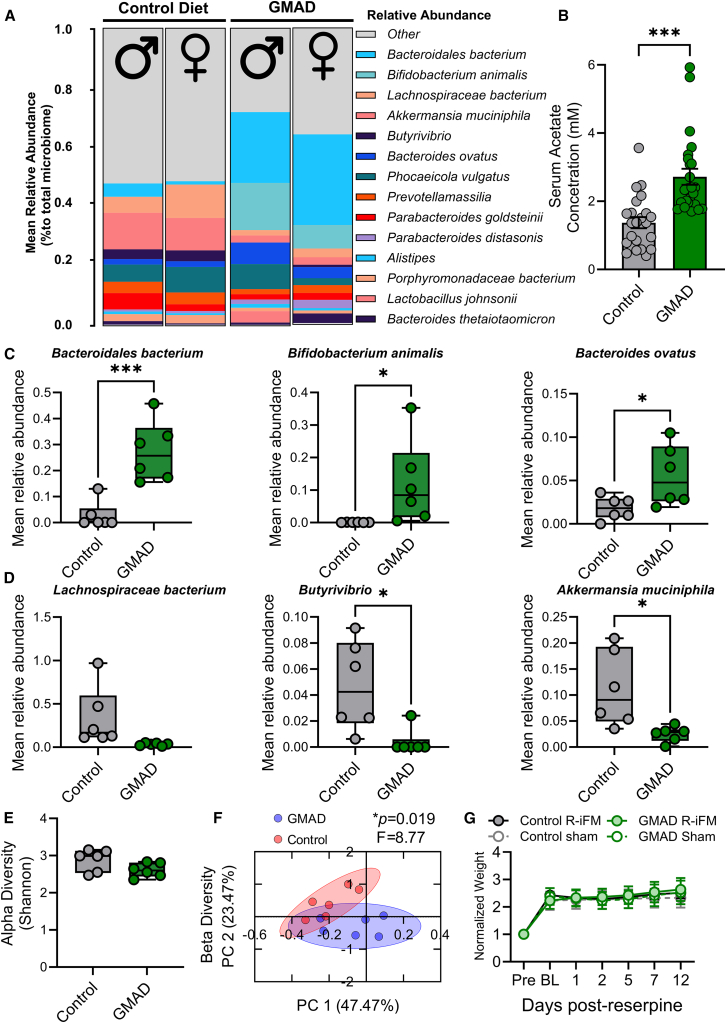
GMAD changes the bacterial composition in the gut microbiome (A) Relative abundance of bacterial genera in fecal samples, from control diet and GMAD determined by Shotgun metagenomic sequencing (*n* = 6/group, sham and R-iFM). (B) Serum acetate concentrations (in mM) are significantly higher in the GMAD group on day 13. Data is shown as mean ± SEM. (C) Acetate producing bacteria *Bacteroidales bacterium, Bifidobacterium animalis*, and *Bacteroides ovatus* are increased in GMAD fecal samples, while (D) *Lachnospiraceae bacterium*, *Butyrivibrio*, and *Akkermansia muciniphila* are decreased (E) alpha diversity was measured in ONE CODEX platform. (F) Principal coordinates analysis (PCoA) based on weighted UniFrac distances shows the beta-diversity of the fecal microbiota in rats. The plot visualizes the overall microbial community composition, 95% confidence ellipses were drawn around the centroid of each treatment group with each point representing a single sample. PERMANOVA analysis indicates a significant difference in microbial community structure between the groups (*p* = 0.019; pseudo-F = 8.77). (G) Body weight showed no significant differences. The microbiome was analyzed at genus-level resolution; statistical significance was determined by a two-tailed *t* test for (B)–(E). ∗*p* < 0.05, ∗∗∗*p* < 0.001.

Having established the effects of GMAD on pain behavior, we next investigated changes in gut microbe composition. This was achieved through shotgun metagenomic sequencing performed on fecal samples that had been collected from control and reserpine-treated rats after 6 weeks on each diet. A significant shift in the gut microbial community was seen in the R-iFM GMAD group, with a transition from a microbiome enriched in butyrate producers to one dominated by acetate-producing taxa, in both male and female animals (Figure 2A). In particular, *Bacteroidales bacterium*, which was present at very low levels in the control group (2.69 ± 0.02%) became one of the most dominant taxa in the GMAD group, with a relative abundance of 27.23 ± 0.05% (Figure 2C; *p* < 0.001). Similarly, the diet significantly promoted the growth of *Bifidobacterium animalis* (from 0.07 ± 0.02% to 11.92 ± 0.05%, *p* = 0.04) and *Bacteroides ovatus* (from 1.81 ± 0.55% to 5.53 ± 1.42%, *p* = 0.03; Figure 2C), both of which are known for their capacity to ferment complex carbohydrates into acetate.^20^^,^^21^^,^^22^^,^^23^^,^^24^^,^^25^ No significant differences in gut microbiome composition were detected in saline control and reserpine-treated of rats on the control diet (Figures S2A and S2B). This suggests that the development of pain in R-iFM, which is known to be caused by biogenic amine depletion,^26^ does not depend on microbiome changes during the first 12 days following reserpine administration.

To determine whether the GMAD diet increased systemic acetate, we measured concentrations in serum samples collected at the experimental endpoint on day 13, which showed significantly higher circulating levels of acetate (2.7 ± 0.23 mM) compared to those on the control diet (1.37 ± 0.16 mM, *p* < 0.001, two-tailed *t* test) (Figure 2B). There was no significant difference between males and females, or between R-iFM and control groups on each diet (Figure S2C). This suggests that the change in microbiome, induced by GMAD, alters systemic acetate levels.

Interestingly, the key butyrate-producing bacteria, including *Lachnospiraceae bacterium* and *Butyrivibrio*, were reduced in the GMAD group (by 33.37 ± 0.14% to 3.34 ± 0.01% and from 4.69 ± 0.01% to 0.40 ± 0.004%, *p* < 0 0.05, respectively; Figure 2D). Furthermore, the abundance of *Akkermansia muciniphila*, a key mucin-degrading specialist, was also significantly reduced from 11.11 ± 0.03% to 0.03 ± 0.01%, *p* = 0.017). Taken together, these data demonstrate that GMAD changes the gut ecosystem to an acetate-producing dominated microbiome, primarily from the *Bacteroidales* order, while simultaneously suppressing key butyrate producers and other resident bacteria that are prevalent in rats fed the control diet. Alpha-diversity analysis showed that both diet groups had similar species diversity (Figure 2E), while analysis of beta-diversity shown a significant shift in the composition of the gut microbiome (*p* = 0.019, F = 8.77; Figure 2F). Furthermore, no significant change was observed in animal weight throughout the experiment (Figure 2G).

### GMAD alters the expression of cytokine mRNA in pain pathways

To understand the mechanisms underlying the GMAD-induced analgesic effects, we investigated changes in gene expression in key sensory processing regions, the DRG and the lumbar spinal cord. Initial tests were performed on DRG using Qiagen RT^2^ Profiler PCR Array Rat Pain: Neuropathic & Inflammatory (cat # 330231), to screen for gene expression changes in 84 targets involved in conduction of pain (ion channels and receptors), synaptic transmission, neuroinflammation, and pain response modulation. Surprisingly, the most notable changes in gene expression among the four groups (*n* = 3 animals/group) were limited to a small group of cytokines, including IL-1α, IL-1β, IL-2, and IL-10 (data not shown). To confirm these preliminary findings and further understand the changes in expression of these neuromodulatory cytokines, we used RT-qPCR to detect transcripts change in both spinal cord and DRG expression of IL-10, IL-1α, IL-1β, IL-2, and IL-6 and in tissues from *n* = 8 animals/group.

In the DRG, the mRNA expression of IL-10, an anti-inflammatory cytokine with neuroprotective and antinociceptive properties, was increased in the GMAD, R-iFM group. Compared with the R-iFM rats on control diet, with IL-10 gene expression of 0.64 ± 0.12 relative to the sham group, the GMAD R-iFM group showed a ∼3-fold increase in expression (2.22 ± 0.61, *p* = 0.024; Figure S1A). No significant changes were seen with IL-1α, IL-1β, IL-2 gene expression in the DRG. A similar shift in cytokine expression was seen in the lumbar spinal cord, with a significant increase in the gene expression of IL-10 (from 1.05 ± 0.30 to 2.83 ± 0.46 in control diet and GMAD R-iFM models, respectively, *p* = 0.004; Figure S1B). A similar, but non-significant, upward trend was also observed in the GMAD sham group. Furthermore, IL-2 expression was also significantly elevated in the spinal cord of the GMAD R-iFM group, with a ∼3.5-fold increase in IL-2 mRNA levels compared to the control-diet sham group (from 1.04 ± 0.17 to 3.41 ± 0.66, *p* = 0.029; Figure S1B). Lastly, the anti-inflammatory cytokine, IL-6, also showed significantly increased mRNA with GMAD in both the sham (4.35 ± 0.56, *p* = 0.005) and R-iFM groups (4.49 ± 1.19, *p* = 0.036), compared with the diet control sham group (1.16 ± 0.25; Figure S1B). These findings suggest that GMAD modulates cytokine gene expression in both DRG and spinal cord, which may contribute to reduced fibromyalgia-like pain.

### GMAD suppresses microglial activation in the spinal cord dorsal horn

Given that GMAD shifted the expression of cytokines in the spinal cord toward an anti-inflammatory state, we next investigated its impact on microglia, the resident immune cells of the CNS. Spinal microglial activation is a hallmark of neuroinflammation and a key cellular mechanism driving the central sensitization that underlies chronic pain states, including in fibromyalgia.^15^ We therefore hypothesized that GMAD may alter the activation state of microglia in the spinal cord dorsal horn. To examine microglia activation states, we performed morphological analysis of Iba1-positive microglia, through a semi-automated, high-throughput analysis using the MicrogliaMorphology plugin^27^ for ImageJ. This allowed us to characterize 27 distinct morphological features, which were used to classify individual microglia into four primary states: one resting (ramified) and three activated (hypertrophic, amoeboid, and rod-like) morphologies. In their resting state, microglia exhibit a ramified morphology with fine, branching processes. Upon activation, they undergo a distinct transformation to a hypertrophic or amoeboid state, characterized by an enlarged cell body and thickened, retracted processes.^28^

**Figure 3:**
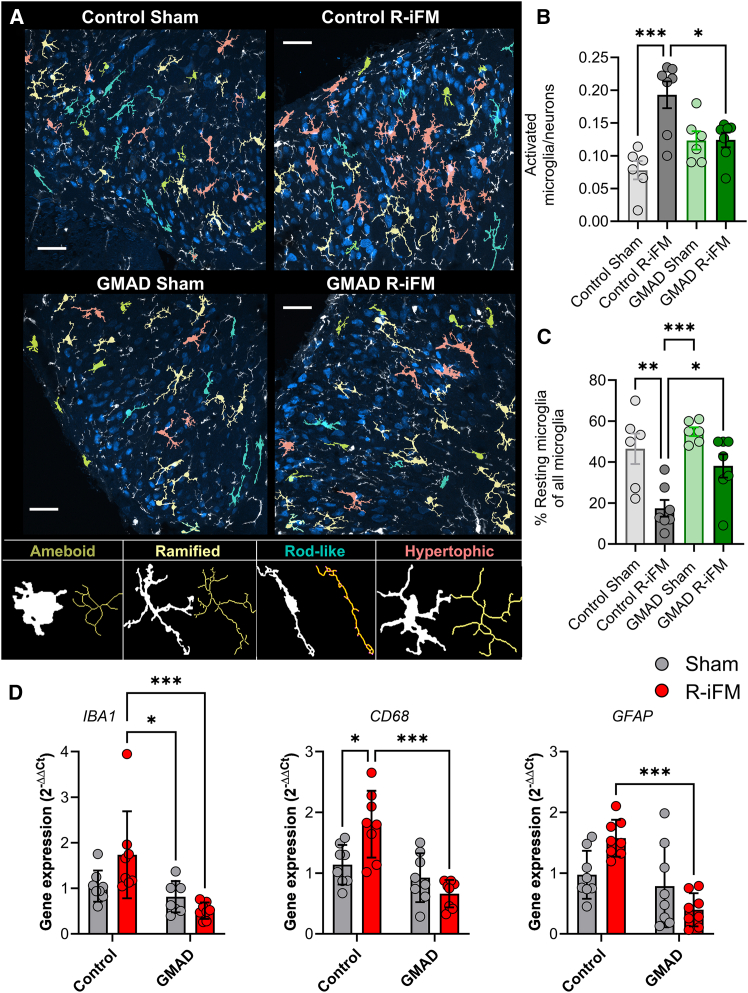
GMAD prevents microglial activation in the spinal cord dorsal horn (A) Representative images of the lumbar dorsal horn show microglial morphology. Iba1-positive microglia are pseudo-colored to indicate their activation state: activated/amoeboid (mustard) resting/ramified (yellow), activated/rod-like (cyan), and activated/hypertrophic (red). Neurons are labeled for NeuN (dark blue). Examples of single-microglia images and computed skeleton line for analysis are shown. (B) The ratio of activated microglia (hypertrophic, amoeboid, and rod-like morphologies combined), to total neurons in GMAD-fed rats compared to control-diet rats. (C) The percentage of resting microglia (expressed as % of total microglia) is increased in the GMAD group, indicating a shift toward a quiescent state. Data are expressed as mean ± SEM, *n* > 6 per group, ≥3 animals in each sex). Statistical significance was determined by a one-way ANOVA followed by Tukey’s post hoc test. ∗*p* < 0.05, ∗∗*p* < 0.01, ∗∗∗*p* < 0.001. Scale bar indicates 40 μM. (D) RT-qPCR data show expression of Iba1 (pan-microglial marker), CD68 (microglial activation/phagocytic marker), and GFAP (reactive astrocyte marker) increased in R-iFM control diet group, compared with GMAD R-iFM group. Data are presented as fold change relative to the control sham group, mean ± SEM. Statistical significance was determined by two-way ANOVA followed by Turkey’s post hoc test, ∗*p* < 0.05, ∗∗∗*p* < 0.01.

In line with the development of pain-like behaviors, rats in the control-diet R-iFM group displayed microglial activation in the spinal cord dorsal horn (Figure 3A). Compared to the ramified (resting) microglia observed in sham controls (Figure 3A), the control-diet R-iFM exhibited a significant shift toward activated, hypertrophic morphologies (Figure S4). Quantitative analysis confirmed these observations, revealing a significant increase in the ratio of activated microglia to neurons (from 0.08 ± 0.01 to 0.19 ± 0.02, *p* < 0.001; Figure 3B) and a corresponding significant decrease in the percentage of resting microglia (from 46.51 ± 7.45% to 17.47 ± 4.06%, *p* = 0.007; Figure 3C) compared to the control-diet sham group. Interestingly, GMAD completely prevented the reserpine-induced microgliosis, and microglia in the GMAD R-iFM group maintained a predominantly quiescent, ramified morphology, appearing indistinguishable from the sham controls (Figure 3C). This neuroprotective effect was quantified which showed GMAD significantly reduces the proportion of activated microglia (0.12 ± 0.01% vs. 0.19 ± 0.02, *p* = 0.02; Figure 3B) and significantly increased the percentage of resting microglia (38.12 ± 0.06% vs. 17.47 ± 4.06%, *p* = 0.04; Figure 3C) when compared to the control-diet R-iFM group.

To validate these morphological findings at the molecular level, we performed RT-qPCR for key microglia activation markers. Induction of R-iFM significantly increased the expression of the microglial marker *IBA1* (from 1.04 ± 0.12 to 1.74 ± 0.33, *p* = 0.07; Figure 3D) and the microglial activation/phagocytic marker *CD68* (from 1.13 ± 0.12 to 1.81 ± 0.19, *p* = 0.011; Figure 3D). GMAD suppressed the expression of both markers (0.51 ± 0.06, *p* < 0.001 and 0.66 ± 0.08, *p* < 0.001, respectively) compared with control R-iFM group, returning them to levels comparable to the sham controls. Furthermore, we found that the GMAD also significantly prevented fibromyalgia model induced *GFAP* upregulation, (from 1.58 ± 0.11 to 0.39 ± 0.09, *p* < 0.007; Figure 3D), reducing *GFAP* expression to baseline levels.

These results suggest that the analgesic effect of the GMAD is associated with the suppression of neuroinflammation in the spinal dorsal horn. By maintaining microglia in a homeostatic, non-reactive state, this diet may prevent the release of pro-nociceptive mediators that contribute to the maintenance of central sensitization, providing a direct cellular link between the diet, the altered cytokine environment, and the attenuation of fibromyalgia-like pain.

### GMAD rescues glycinergic inhibition in spinal cord superficial laminae neurons

#### GMAD reduces the intrinsic excitability of dorsal horn neurons

To investigate the effects of the GMAD diet on neuronal activity of nociceptive neurons in the R-iFM model, we performed whole-cell patch-clamp recordings from lamina I/II neurons in the spinal dorsal horn, a region involved in the processing and transmission of nociceptive signals. We show that the R-iFM model induces a state of neuronal hyperexcitability in dorsal horn neurons, which is reversed by the GMAD through actions on both the intrinsic properties of neurons and the balance of synaptic input they receive.

**Figure 4:**
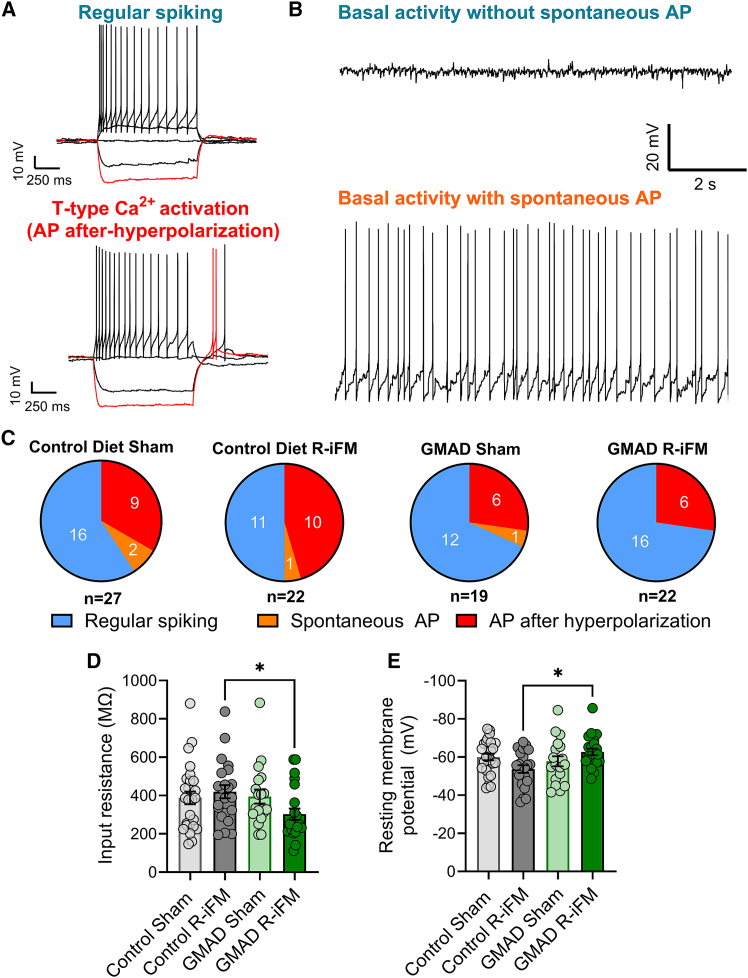
GMAD reduces neuronal excitability in the spinal cord dorsal horn (A) Representative whole cell current-clamp recordings showing neuronal responses to a series of hyperpolarizing and depolarizing current steps in control sham and R-IFM neurons. We classified firing types into three broad groups: (1) regular firing on depolarization, with no spontaneous activity, (2) spontaneous firing with no AP-after hyperpolarization, (3) AP after-hyperpolarization. (B) Representative traces of basal activity with and without spontaneous action potential firing. (C) Pie charts showing the distribution of neuronal firing phenotypes across the four experimental groups, with total number of recorded neurons indicated for each group. (D) Neuronal input resistance shows a significant decrease in the GMAD R-iFM group compared to control. (E) Resting membrane potential is hyperpolarized in the GMAD R-iFM group compared to the control R-iFM group. Data are presented as mean ± SEM. Statistical significance was determined by a one-way ANOVA with Tukey’s post hoc test (∗*p* < 0.05).

**Figure 5:**
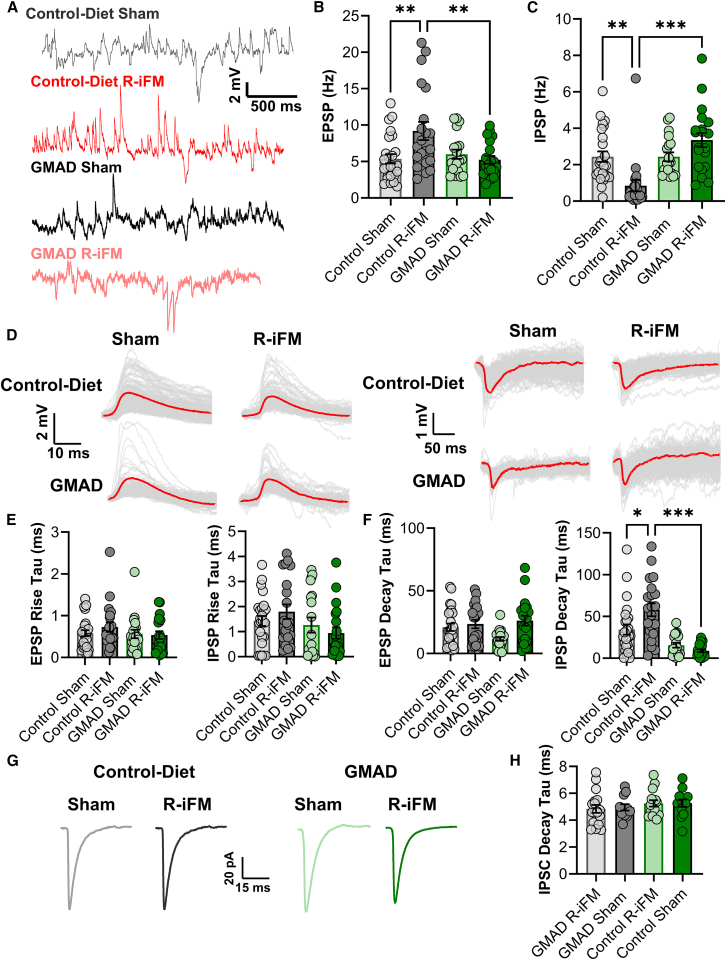
GMAD restores synaptic balance in lamina I/II neurons by attenuating excitatory signals and rescuing inhibitory activity (A) Representative traces of spontaneous excitatory and IPSPs, measured in whole-cell current clamp from lamina I/II neurons in the spinal cord dorsal horn. (B and C) Quantification of spontaneous synaptic potentials shows that control-diet R-iFM animals have significantly higher EPSP frequency and lower IPSP frequency, compared to the sham control-diet. Frequency of both IPSPs and EPSPs is restored to control levels in the GMAD R-iFM group. (D) Traces show EPSPs and IPSPs events (>100 per analysis) detected from representative recordings, with average (overlayed, red) from all four groups. (E) Quantification of the rise tau values for EPSPs and IPSP show no difference across treatment groups. (F) The EPSC decay tau show no difference across groups; while decay tau for IPSCs shows the control-diet R-iFM group have significantly higher tau, compared with the sham and GMAD R-iFM groups. (G) Pharmacologically isolated glycine IPSCs were recorded under voltage-clamp, in the presence of NBQX, APV, and gabazine (GBZ). (H) The average decay tau shows no significant differences across four groups (*n* > 10 neurons from >3 animals in each group). For (B), (C), (E), and (F), recordings were taken from >18 neurons from >3 animals of each gender per group. Data are presented as mean ± SEM, with individual data points shown. Statistical significance was determined by a one-way ANOVA followed by Tukey’s post hoc test. ∗*p* < 0.05, ∗∗*p* < 0.01, ∗∗∗*p* < 0.001.

We first characterized the firing patterns of lamina I/II neurons in response to current injections, into three broad groups including: (1) regular spiking, which includes all firing types seen in the dorsal horn (including phasic, delayed, single spike, and tonic), (2) spontaneous action potential firing with no hyperpolarization-induced action potentials, and (3) action potential after hyperpolarization (characterized by a burst of action potentials following a hyperpolarizing current step) with or without basal spontaneous activity (Figure 4A). We found that some neurons exhibited both spontaneous firing and AP-after hyperpolarization, across all four groups: GMAD sham (*n* = 2), GMAD R-iFM (*n* = 2), control-diet sham (*n* = 3), and control-diet R-iFM (*n* = 2). Since T-type calcium channel activation is a key regulator that drives neuronal hyperexcitability, and spontaneous action potentials are a primary indicator of this hyperexcitable state, we classified these neurons exhibiting both of these characteristics in the AP after hyperpolarization group. In R-iFM animals, the GMAD intervention decreased the percentage of neurons exhibiting the T-type calcium channel activation phenotype to 27.27% (6 of 22 neurons), down from 45.45% (11 of 22 neurons) in animals on the control diet (Figure 5C). This reduced level is comparable to that observed in the sham groups on both the GMAD (31.58%) and control diets (33.33%).

Consistent with central sensitization, we found that the intrinsic properties of superficial laminae neurons were altered in the control R-iFM group, with increased excitability, as shown by increased input resistance and depolarized resting membrane potential (Figures 4D and 4E). Interestingly, GMAD caused a significant hyperpolarization in resting membrane potential to in the R-iFM group compared with control diet (from −53.68 ± 2.01 mV to −62.24 ± 1.74 mV, *p* = 0.016; Figure 4D), restoring it to a level that was not significantly different from sham controls (−58.80 ± 2.92 mV, *p* = 0.66; Figure 4F), thereby reducing hyperexcitability. This was accompanied by a significant reduction in input resistance, from 419.6 ± 34.48 MΩ in control-diet to 302.6 ± 29.36 MΩ in the GMAD R-iFM group (*p* = 0.04; Figure 4E), leading to decreased excitability of these neurons. Together, these changes effectively reset the neurons intrinsic excitability, providing a mechanism to counteract the sensitized state.

#### GMAD restores inhibitory synaptic activity

In addition to increased intrinsic excitability of dorsal horn neurons, we also found that the frequency of miniature and spontaneous excitatory postsynaptic potentials (EPSPs) were significantly increased in the R-iFM control diet group (9.20 ± 1.24 Hz) compared to sham controls (5.37 ± 0.63 Hz to, *p* = 0.005; Figures 5A and 5B), indicating increased excitatory synaptic activity within these circuits. We found that GMAD reduced sEPSP frequency significantly (5.24 ± 0.54 Hz, *p* = 0.005; Figures 5A and 5B) compared with control-diet R-iFM, restoring the excitatory/inhibitory activity within nociceptive circuits. This was accompanied by a ∼70% decrease in the frequency of spontaneous inhibitory postsynaptic potentials (IPSPs), from 2.44 ± 0.29 to 0.84 ± 0.31 Hz, *p* = 0.002 (Figure 5C), suggesting that the control-diet R-iFM group had reduced synaptic inhibition, a characteristic of pathological pain. We found that GMAD reversed the loss of inhibitory synaptic signaling, restoring the sIPSP frequency in the GMAD R-iFM group to 3.35 ± 0.39 Hz, which was not significant to the sham control GMAD neurons (2.43 ± 0.25 Hz, *p* = 0.114, Figures 5A and 5C).

Further analysis revealed changes in inhibitory synaptic potential kinetics, where the R-iFM group on control-diet showed an sIPSP decay time constant (decay Tau) that was significantly higher than the sham group (57.97 ± 7.71 ms compared to 34.22 ± 6.12 ms, *p* = 0.011; Figures 5D and 5F). GMAD significantly reduced the sIPSP decay time in the R-iFM group to 8.89 ± 1.71 ms (*p* < 0.001 vs. control-diet R-iFM; Figures 5D and 5F), which may suggest a change in inhibitory receptor subtype, as previously reported.^29^ In contrast, the EPSP decay Tau showed no significant variation across any group (Figures 5D and 5F). Additionally, no significant differences were seen in rise tau, in either sEPSP or sIPSP, between the two diets and both sham and R-iFM conditions (*p* > 0.05 in all comparison; Figure 5E). Further experiments in the presence of NBQX, APV, and gabazine (GBZ), to pharmacologically isolate spontaneous glycine inhibitory synaptic currents (sIPSCs) in a voltage clamp configuration, showed no significant difference in decay tau across all groups (Figures 5G and 5H), which suggests that the glycinergic currents with faster decay are being suppressed in the R-iFM state, leaving the relatively slower, GABAergic-dominant inhibitory currents intact. These data suggest that GMAD may reduce intrinsic excitability of dorsal horn neurons and rescue synaptic inhibition. These data provide a direct functional mechanism for the behavioral effects of GMAD, which may effectively dampen the central sensitization that underlies and maintains the chronic pain state in the R-iFM model.

#### GMAD prevents PGE2-mediated suppression of glycinergic inhibition

**Figure 6:**
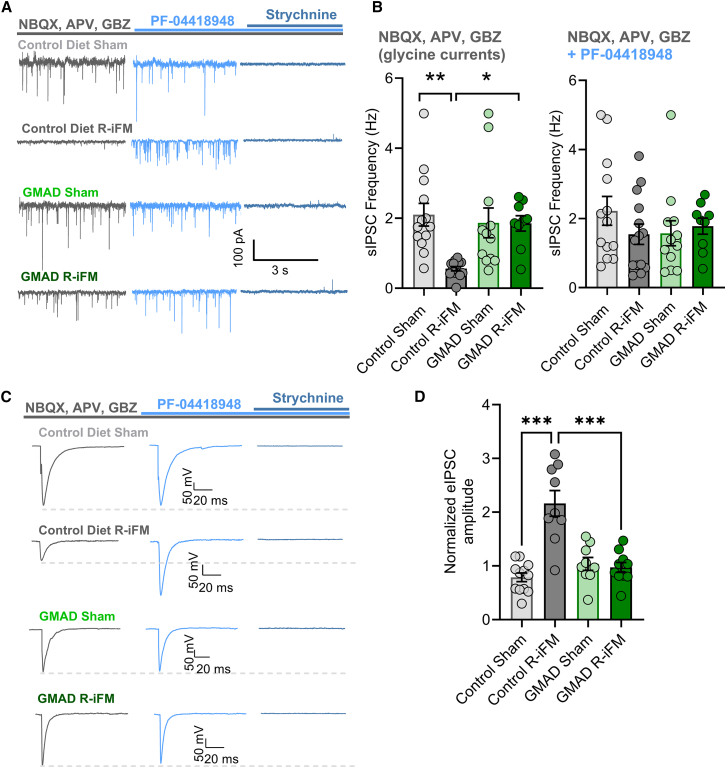
GMAD rescues glycinergic inhibition from PGE2-mediated suppression (A) Representative traces show spontaneous glycinergic currents recorded from dorsal horn neurons in voltage-clamp in the presence of NBQX, APV, and gabazine (GBZ) (blue), with the EP2 receptor antagonist PF-04418948 (red), and inhibited by the glycine receptor antagonist strychnine (dark blue trace). (B) Quantification of sIPSC frequency prior to and after PF-04418948 application, shows significantly reduced frequency of events in the control-diet R-iFM group, compared to the control-diet sham group. This was not observed in the GMAD groups, which had similar frequencies. Bath application of strychnine, a glycine receptor antagonist, abolished all remaining sIPSC events across all groups, confirming the events were glycinergic (*n* = 6 animals/group with equal number of males and females, from >9 neurons per group, at a holding potential of −70 mV). (C) Example traces of evoked glycinergic IPSCs which show the addition of PF-04418948 significantly increases eIPSC amplitude in the control-diet R-iFM group. (D) Quantification of normalized eIPSC amplitude (% of baseline). Glycinergic inhibition is increased by PF-04418948 in the control-diet R-iFM group but unchanged in the GMAD groups (*n* = 6 animals/group with equal number of males and females, from >15 neurons per group, at a holding potential of −70 mV). Data are presented as mean ± SEM. Statistical significance was determined by a one-way ANOVA followed by Tukey’s post hoc test. ∗*p* < 0.05, ∗∗*p* < 0.01, ∗∗∗*p* < 0.001.

As our data suggests that GMAD restores inhibitory synaptic activity, which appears to be due to a restoration of glycinergic activity, in addition to its effect of preventing microglial activation, we hypothesized that these effects may be mediated by the prostaglandin E2 (PGE2). Activated microglia are known to release PGE2,^30^ which specifically suppresses glycinergic neurotransmission in the spinal cord by activating the EP2 receptor.^31^ To test this hypothesis, we first pharmacologically isolated the glycinergic component of the evoked IPSC. In all groups, non-glycinergic synaptic activity was blocked by a cocktail of NBQX, APV, and GBZ, and the remaining current was confirmed to be glycinergic by its complete inhibition with the glycine receptor antagonist strychnine (Figure 6A). Recordings showed that the frequency of spontaneous glycinergic currents was significant lower in the R-iFM control-diet group (0.55 ± 0.06 Hz), compared with the sham group on control-diet (2.10 ± 0.32 Hz, ∗∗∗*p* < 0.001; Figure 6B). Furthermore, the EP2 receptor antagonist PF-0441894 recovered sIPSC frequency in the R-iFM control-diet group, 0.55 ± 0.06 to 1.54 ± 0.29 Hz (Figure 6B), bringing it to the same level as other groups (2.22 ± 0.41 Hz, 1.57 ± 1.25 Hz, and 1.78 ± 0.23 Hz, control sham, GMAD sham, and GMAD R-iFM, respectively); no significant change can be seen between other groups.

Similarly, in recordings of evoked glycinergic inhibitory postsynaptic currents (eIPSCs) from dorsal horn neurons following electrical stimulation of deeper lamina, the current amplitude in neurons from the control-diet R-iFM group increased significantly following application of the EP2 receptor antagonist PF-04418948. These currents were again completely inhibited by the glycine receptor antagonist strychnine, confirming their glycinergic nature (Figure 6C). Compared to sham controls, the normalised current increased from 0.78 ± 0.08 to 2.16 ± 0.24, *p* < 0.001 in the control diet group (Figure 6D), suggesting a state of glycinergic disinhibition consistent with central sensitization. Application of PF-04418948 to either sham injection groups on either control or GMAD diets shows no significant change in eIPSC amplitude (0.79 ± 0.08 and 1.04 ± 0.12, respectively, *p* = 0.484; Figure 6D), suggested that the glycinergic current is not suppressed in a non-fibromyalgia-like state. Importantly, the suppression of glycinergic neurotransmission was completely absent in rats fed GMAD. The GMAD R-iFM group maintained glycinergic eIPSC amplitudes that were unaffected by the application of PF-04418948 compared with its sham group (0.97 ± 0.09 and 1.03 ± 0.11, respectively, *p* = 0.745; Figure 6D). This supports the hypothesis that glycinergic inhibitory activity is supressed in the R-iFM model, but rescued in the group fed the GMAD diet.

Collectively, these findings provide a potential mechanistic link between the gut microbiome, neuro-inflammation, and synaptic function. GMAD prevents spinal microglial activation, which in turn prevents the pathological release of PGE2. By averting the subsequent suppression of glycinergic inhibition, the diet preserves spinal inhibitory activity, counteracts central sensitization, and ultimately alleviates pain-like behaviors.

## Discussion

Fibromyalgia is a complex and debilitating chronic pain syndrome characterized by widespread pain, fatigue, and cognitive disturbances. Its pathophysiology remains elusive, but growing evidence points to central sensitization and neuroinflammation as key underlying mechanisms.^4^^,^^32^^,^^33^ It has also become increasingly recognized that gut-spinal axis acts as a critical regulator of host physiology, with gut microbial metabolites influencing neurological and immune functions that may contribute to this neuroinflammatory process. In this study, we establish a potential mechanistic link between a gut-derived metabolite, acetate, and the attenuation of fibromyalgia-like symptoms. Although R-iFM is a model with different etiology to fibromyalgia, it mimics symptoms that patients report, including touch sensitivity and depression,^26^ but does not replicate the long-term changes in gut microbiota.^15^ Nevertheless, here we demonstrate that a targeted diet can induce a shift in the gut microbiome toward acetate production, which elevates systemic acetate levels, and suppresses neuroinflammation from the peripheral sensory ganglia to the spinal cord. By preventing microglial activation,^11^^,^^34^ systemic acetate acts to restore spinal inhibitory neurotransmission, ultimately reversing the central sensitization that drives chronic pain in this rodent model of fibromyalgia.

Here, we show that the GMAD-based diet induces a taxonomic and functional shift in the microbiome, from a community dominated by butyrate- and propionate-producing bacteria, such as *Lachnospiraceae* and *Butyrivibrio*, to one dominated by potent acetate producers. This is supported by previous research that shows acetylated high amylose starch diets, preferentially increase systemic acetate, rather than butyrate.^35^ Specifically, we observed a significant enrichment of taxa within the *Bacteroides* order, including *Bacteroidales bacterium*, *Bacteroides ovatus*, and *Phocaeicola vulgatus*, as well as *Bifidobacterium animalis,* which are known to produce acetate.^21^^,^^22^^,^^23^^,^^24^^,^^25^^,^^36^ Increased acetate levels would be expected in this microbiome, as bacteria of the *Bacteroides* order are specialists in degrading complex carbohydrates, through excretion of glycosyl hydrolases that break down plant-based resistant starches that are indigestible to the host.^35^^,^^37^ Indeed, diets high in resistant starch have been shown to increase the abundance of *Bacteroides* species.^38^ An increase in *Bifidobacterium animalis*, which is known to digest fermentable fibers^39^ using a unique metabolic pathway known as the “bifidus shunt” will also contribute to the production of high levels of acetate and lactate.^40^ Interestingly, the diet not only promoted acetate production but also appeared to inhibit its subsequent conversion to butyrate. The control diet fostered a community rich in *Lachnospiraceae* and *Butyrivibrio*, bacteria known to convert acetate into butyrate via the butyryl-CoA:acetate CoA-transferase pathway. The GMAD dramatically reduced the abundance of these taxa, with *Lachnospiraceae* decreasing from 11.55% to 2.88% and *Butyrivibrio* to less than 1%. Through this dual mechanism, enhancing production while simultaneously blocking conversion, this dietary strategy successfully increased systemic acetate levels, confirming that gut-derived metabolites can exert effects on distal systems like the CNS.

The capacity of systemically elevated acetate to drive anti-neuroinflammatory effects is well-supported, and acetate is known to abrogate increases in IL-1β in models of neuroinflammation.^34^ Moreover, acetate promotes an anti-inflammatory state by upregulating the expression of suppressive cytokines, through its function as an inhibitor of class I and II HDACs, an action known to enhance IL-10 expression.^41^^,^^42^^,^^43^ Previous literature has demonstrated that fibromyalgia is associated with elevated pro-inflammatory cytokines such as IL-1α, IL-1β, IL-6, IL-8, and IL-18 in peripheral circulation.^7^^,^^8^ However, some studies have also reported decreased or no change in circulating IL-1α and IL-1β in fibromyalgia patients, this is often accompanied by significantly higher levels of the soluble receptor IL-1Ra, suggesting a complex regulatory environment.^44^^,^^45^^,^^46^ Our findings align with this complexity, as our fibromyalgia model did not induce a significant transcriptional upregulation of the proinflammatory cytokines IL-1α or IL-1β in the DRG (Figure S1A). However, the most striking finding in the periphery was not the absence of inflammation, but rather the induction of an anti-inflammatory response in the GMAD group. Specifically, the diet induced a significant upregulation of gene expression of the canonical anti-inflammatory cytokine IL-10 in the DRG of fibromyalgia animals (Figure S1A). This suggests that a primary mechanism of the diet’s peripheral action is not to reverse an existing inflammatory state, but to create a “pro-resolution shield” at the level of the primary sensory neuron, which likely prevents the initiation and propagation of nociceptive signals to the spinal cord.

This anti-inflammatory effect extends centrally into the spinal cord, where the diet prevented the activation of microglia, a key cellular driver of central sensitization (Figure 3). In our reserpine-induced fibromyalgia model, we found a decrease in spinal microglia with the resting ramified morphology (Figure 3C) and an increase in the activated hypertrophic morphology (Figure S4). This activated state of microglia has been reported previously in fibromyalgia models^15^^,^^47^ and is an established signature of neuroinflammation causally linked to the maintenance of chronic pain.^48^ GMAD completely suppressed this microgliosis, which is strongly correlated with the diet’s analgesic effect, as activated microglia are a major source of pro-nociceptive mediators that amplify pain signaling within the dorsal horn.

The reduction in microglial activation observed in this study, is likely to be a result of the synergistic mRNA upregulation of IL-2, IL-6, and IL-10 (Figures S1A and S1B). Previous studies in fibromyalgia patients have reported varying levels of systemic IL-10^9^^,^^44^^,^^46^^,^^49^ while our study shows similar expression of IL-10 in both DRG and spinal cord in control and R-iFM states on a control diet, with a significant increase in both groups on GMAD (Figure S1B). In many chronic pain models, IL-6, much like IL-1β and TNF-α, is considered a key pro-inflammatory and pro-nociceptive mediator, however, the function of IL-6 is highly context-dependent (e.g., it is increased in exercise^50^), and it can also exert anti-inflammatory and neurotrophic effects. Furthermore, it was suggested that in CNS, IL-6 exerts a neuroprotective effect by inhibiting neuronal apoptosis and inflammatory mediators.^51^ In the anti-inflammatory environment created by GMAD, characterized by quiescent microglia, the observed increase in IL-6 mRNA expression likely reflects its contribution to a pro-resolution, rather than a pro-inflammatory state. While reports on IL-2 expression in fibromyalgia have also been inconsistent,^46^^,^^52^ the upregulation of IL-2 gene expression in our GMAD group is a particularly novel finding, as there is currently no evidence *in vivo* that suggests circulating acetate can increase IL-2 expression. This effect may be due to the action of acetate on the differentiation of T cells into T helper 1 (Th1) cells, which are then further stimulated by acetate to secrete IL-2.^53^ This dual enhanced transcription in IL-10 and IL-2 is highly significant as both cytokines are critical for the function of immunosuppressive regulatory T cells (Tregs). IL-2 is essential for the development, maintenance, and function of Tregs,^54^ while IL-10 can also induce their differentiation and enhance their capabilities.^55^ This is highly relevant, as studies have shown a reduction in Treg populations in fibromyalgia patients.^56^ Tregs are key players in pain resolution and can directly inhibit pain by suppressing spinal microglia activation.^57^^,^^58^ Furthermore, Tregs themselves are a major source of IL-10,^59^ suggesting the establishment of an anti-inflammatory cycle. Based on evidence from these previous studies, we propose that the acetate-induced increases in mRNA expression in both IL-2 and IL-10, may contribute to a neuro-immune environment that supports and enhances Treg populations. These Tregs, in turn, actively prevent microglia from activating, thereby amplifying and stabilizing the anti-inflammatory state within the spinal cord and preventing the development of neuronal hyperexcitability.

Lastly, this combination of neuroimmune changes appear to converge, which normalizes synaptic function within spinal pain circuits. Our electrophysiological recordings reveal a state of central sensitization in the reserpine model, characterized by intrinsically hyperexcitable dorsal horn neurons that exhibited increased rate of firing and a depolarized resting membrane potential (Figure 4). We also observe hyperpolarization-activated action potentials, which are characterized by a depolarization from the hyperpolarized membrane potential, (Figures 4A and 4B) and represent a state of intrinsic hyperexcitability.

The increased neuronal burst firing observed in spinal cord from our fibromyalgia model is likely driven by the dysregulation of low-voltage-activated T-type calcium channels, specifically the CaV3.2 isoform. These channels are pivotal regulators of neuronal excitability because they activate at membrane potentials near the resting state.^60^ Following an initial action potential, the activation of T-type channels generates a sustained inward calcium current, producing a prolonged afterdepolarization (ADP). This ADP can overcome the after hyperpolarization period, bringing the neuron back to threshold and triggering a high-frequency burst of action potentials; a mechanism that directly explains the firing phenotype we observed (Figure 4A). The upregulation of T-type channel expression and function in the spinal dorsal horn is a well-established hallmark of the neuronal hyperexcitability that underlies central sensitization in chronic inflammatory and neuropathic pain.^60^ The critical role of CaV3.2, in particular, has been demonstrated in a mouse model of fibromyalgia-like chronic musculoskeletal pain, where CaV3.2-deficient mice did not develop chronic mechanical hyperalgesia, and pharmacological blockade of the channel reversed pain.^61^ This would suggest that the prevention of this pathological firing by GMAD may protect against the development of central sensitization, in reserpine-induced fibromyalgia rat model (Figure 4D). GMAD appears to reverse the pathological state, restoring normal firing phenotypes and membrane properties, likely driven by the reduction of neuroinflammation. In this fibromyalgia model, we observe an increase in EPSC frequency and a reduction in IPSC frequency, which was reversed by GMAD, via an increase in inhibitory neuronal activity (Figures 5B and 5C). Further kinetic analysis revealed a slowing of IPSC decay time in the fibromyalgia model in control-diet, which we hypothesized reflects a loss of glycinergic inhibition (Figures 5E and 5G). Our final experiment provides a precise molecular and synaptic mechanism for this restored glycinergic inhibition. Activated microglia are known to release prostaglandin E2 (PGE2),^30^ which selectively suppresses glycinergic neurotransmission via the EP2 receptor.^62^^,^^63^^,^^64^ By maintaining microglia in a quiescent state, the acetate diet prevents this pathological release of PGE2, thereby preserving the integrity of glycinergic inhibitory synapses in the dorsal horn (Figure 6). This provides a potential, multi-level mechanistic pathway that links the diet-induced changes in the gut to the functional rescue of nociceptive signaling.

In summary, this study delineates a gut-to-spinal cord axis for pain modulation. Studies have shown that the transplant of gut microbiota from fibromyalgia patients to mice can cause peripheral inflammation and pain.^15^ While other studies have shown that the spinal cord injury can cause gut dysbiosis.^65^ Both studies suggested that the existence of gut-to-spinal cord axis. Here, we are the first to propose a therapeutic model that leverages this. We demonstrate that a specific diet remodels the gut microbiome to favor acetate-producing bacteria, leading to elevated systemic acetate. This metabolite then acts as a powerful signal to modulate the neuro-immune axis, increasing the levels of anti-inflammatory cytokine transcripts in both DRG and spinal cord, which is associated with a reduction in spinal microglial activation. This state corrects the excitatory/inhibitory imbalance and resolves hyperexcitability in dorsal horn lamina I/II neurons. By preserving glycinergic inhibition, the diet normalizes neuronal firing and alleviates fibromyalgia-like pain. These findings highlight the therapeutic potential of targeting the gut microbiome with dietary metabolites as a disease-modifying strategy for complex chronic pain syndromes.

### Limitations of the study

Although we see changes in cytokine expression in DRG and spinal dorsal horn from in rats fed the GMAD diet, this was determined using RT-qPCR, which may not necessarily translate into significant differences in protein concentration. However, these changes we observe have been reported previously in studies using diets that promote acetate production, and they are also known to reduce pain signaling. There may also be microbiome-produced SCFAs other than acetate that influence pain sensitivity, which this study does not investigate. Previous studies using a 15% acetylated high-amylose starch-based diet, show significant increases in serum acetate, with no effect on butyrate or propionate in mice.^39^

## Resource availability

### Lead contact

Requests for further information and resources should be directed to and will be fulfilled by the lead contact, Wendy L. Imlach (wendy.imlach@monash.edu).

### Materials availability

This study did not generate new unique reagents.

### Data and code availability

All data reported in this paper will be shared by the lead contact upon request, or can be accessed in the Mendeley data repository with Mendeley Data: https://doi.org/10.17632/5xz8hk2zxt.1. Metagenomic sequencing can be accessed through NCBI SRA with the BioProject number NCBI: PRJNA1426138.

## Acknowledgments

This work was funded by the Australian 10.13039/501100000925National Health and Medical Research Council (10.13039/501100000925NHMRC grant number 1139586) and the 10.13039/501100000923Australian Research Council (ARC grant number DP250104274) awarded to W.L.I. The authors acknowledge use of facilities within the Monash Micro Imaging.

## Author contributions

Conceptualization, W.L.I.; formal analysis, S.C. and W.L.I.; funding acquisition, W.L.I.; investigation, S.C. and W.L.I.; methodology, W.L.I.; supervision, W.L.I.; writing S.C., D.S., and W.L.I.

## Declaration of interests

The authors declare no competing interests.

## STAR★Methods

### Key resources table

**Table:** 

REAGENT or RESOURCE	SOURCE	IDENTIFIER
**Antibodies**		

Rabbit Anti-IBA1	Abcam	Cat#Ab178847; RRID: AB_2832244
Goat Anti-NeuN	Thermofisher	Cat#PA5143586; RRID: AB_2942815
Donkey Anti-Rabbit Alexa 488	Abcam	Cat#Ab150073; RRID: AB_2636877
Donkey Anti-Goat Alexa 647	Abcam	Cat#Ab150135; RRID: AB_2687955

**Chemicals, peptides, and recombinant proteins**		

Reserpine	Sigma-Aldrich	Cat#83580; CAS: 50-55-5
NBQX	Abcam	Cat#Ab120046; CAS: 479347-86-9
D-APV	Cayman Chemical	Cat#14539; CAS: 79055-68-8
Gabazine	Abcam	Cat#Ab120042; CAS: 104104-50-9
PF-04418948	Tocris	Cat#4818; CAS: 1078166-57-0
Strychnine hydrochloride	Sigma-Aldrich	Cat#S8753; CAS: 1421-86-9

**Critical commercial assays**		

Colorimetric Acetate Assay Kit	Abcam	Cat#Ab204719
RT2 First Strand Kit	Qiagen	Cat#330404
Zirconium Beads	Merck	Cat#Z763799
RNeasy Plus Mini	Qiagen	Cat#74104
RT2 Profiler™ PCR Array Rat Pain: Neuropathic & Inflammatory	Qiagen	Cat#330231

**Experimental models: Organisms/strains**		

Rats: Sprague Dawley	Monash University Animal Research Platform (MARP)	–

**Oligonucleotides**		

RT2 qPCR Primer Assays	Qiagen	Cat#330001
Primer: *Iba* Forward:ATGCTGGAGAAACTTGGGGT Reverse:GCCACTGGACACCTCTCTAATT	LGC Biosearch Technologies	Cat#PP-5
Probe for primer *Iba*: TCCCAAGACCC ATCTAGAGC	LGC Biosearch Technologies	Cat#DLO-RFB-5
Primers: *Cd68* Forward: AGCCATGTGTTCAGCTCCAA Reverse: TTCGGGTTCAATACAGAGAGGC	LGC Biosearch Technologies	Cat#PP-5
Probe for primer *Cd68*: TCGCATCTTG TACCTGACCC	LGC Biosearch Technologies	Cat#DLO-RFB-5
Primers: *GFAP* Forward: GCCACCAGTAACATGCAAGA Reverse: AACGTCTGTGAGGTCTGCAA	LGC Biosearch Technologies	Cat#PP-5
Probe for primer *GFAP*: CAGAAGAGT GGTATCGGTCCA	LGC Biosearch Technologies	Cat#DLO-RFB-5
Primers: *Il-1α* Forward: TGCTCAGGGAGAAGACAAGC Reverse: GGAAAGCTGCGGATGTGAAG	LGC Biosearch Technologies	Cat#PP-5
Probe for primer *Il-1α*: TGTGTTGCTGAA GGAGATTCCGGA	LGC Biosearch Technologies	Cat#DLO-RFB-5
Primers: *Il-1β* Forward: GTGCTGTCTGACCCATGTGA Reverse: GATTCTTCCCCTTGAGGCCC	LGC Biosearch Technologies	Cat#PP-5
Probe for primer *Il-1β*: GCAACGACAAA ATCCCTGTGGC	LGC Biosearch Technologies	Cat#DLO-RFB-5
Primers: *Il-2* Forward: CTCCCCATGATGCTCACGTT Reverse: CAAATCCAACACACGCTGCA	LGC Biosearch Technologies	Cat#PP-5
Probe for primer *Il-2*: CCCAAGCAGGCC ACAGAATTGA	LGC Biosearch Technologies	Cat#DLO-RFB-5
Primers: *Il-10* Forward: TCCCTGGGAGAGAAGCTGAA Reverse: TTCTTCACCTGCTCCACTGC	LGC Biosearch Technologies	Cat#PP-5
Probe for primer *Il-10*: AGCTGCGACGC TGTCATCGA	LGC Biosearch Technologies	Cat#DLO-RFB-5
Primers: *Hprt1* Forward: CTTCCTCCTCAGACCGCTTT Reverse: CACTAATCACGACGCTGGGA	LGC Biosearch Technologies	Cat#PP-5
Probe for primer *Hprt1*: CGAGCCGACC GGTTCTGTCA	LGC Biosearch Technologies	Cat#DLO-RFB-5

**Software and algorithms**		

GraphPad Prism software	Version 10.0, GraphPad Software, Inc.	https://www.graphpad.com/
Clampfit 10 software	Molecular Devices, USA	https://support.moleculardevices.com/
pClamp 10 software	Molecular Devices, USA	https://support.moleculardevices.com/
Fiji	NIH	https://fiji.sc/
MicrogliaMorphology plugin	GitHub	https://github.com/ciernialab/MicrogliaMorphology

**Other**		

GMAD diet	Specialty Feeds (WA, Australia)	Cat#SF21-050
AIN93G diet	Specialty Feeds (WA, Australia)	Cat#SF-AIN93G

### Experimental models and study participant details

#### Animals

Adult male and female Sprague-Dawley rats (6–8 weeks at the start of experiments) were obtained from Monash University animal research platform (MARP). They were housed at 20°C–22°C and 40–60% humidity, with a 12:12 h light/dark cycle and *ad libitum* access to food and water, in appropriate plexiglass cages with wood-shaving bedding and enrichment. All rats allowed to acclimatize for at least 15 min prior to behavioral testing, which were all conducted during the light cycle at the same time each day.

These studies comply with ethical regulations approved by Monash University Animal Ethics Committee (MUAEC-1 approval number: 38782) in accordance with the Australian Code for the Care and Use of Animals for Scientific Purposes (2013). All experimental procedures were conducted following the ARRIVE 2.0 guidelines and according to the ethical principles of the IASP. Investigators were blinded to diet and treatment (reserpine or control) throughout the experiment and treatment groups were randomised with both control and r-iFM animals in each cage.

#### Reserpine-induced fibromyalgia animal models and acetate-producing diet intervention

Reserpine (Sigma-Aldrich) was dissolved in 0.5% acetic acid in saline solution, and subcutaneously injected at 0.8 mg/kg for 3 consecutive days (Day 0 to day 3). Control animals received vehicle (0.5% acetic acid in saline), with the same schedule of administration.

Animals were put on specific diets 28 days before the first injection of reserpine or vehicle to provide sufficient time for change in the microbiota to occur.^35^^,^^66^ An equal number of male and female rats were grouped into either Gut-Microbiota Acetogenic Diet (GMAD) or control diet (AIN93G) (Specialty Feeds (WA, Australia). The GMAD diet contained 15% acetylated high amylose resistant starch, which is consistent with studies by other groups.^35^^,^^39^^,^^66^ The GMAD diet (cat no SF21-050) contains (g/Kg): 200 Casein (Acid), 100 Sucrose, 70 Canola Oil, 50 Cellulose, 386 Wheat Starch, 150 acetylated high amylose resistant starch made from maize starch, 3 L-Methionine, 13.1 Calcium Carbonate, 2.6 Sodium Chloride, 1.4 AIN93 Trace Minerals, 2.5 Potassium Citrate, 6.9 Potassium Dihydrogen Phosphate, 1.6 Potassium Sulfate, 2.5 Choline Chloride (75%) and 10 AIN93 Vitamins; the AIN93G diet, a semi-pure growth diet (cat no, SF-AIN93G) diet contains (g/Kg): 200 Casein (Acid), 100 Sucrose, 70 Canola Oil, 50 Cellulose, 404 Wheat Starch, 132 Dextrinised Starch, 3 L-Methionine, 13.1 Calcium Carbonate, 2.6 Sodium Chloride, 1.4 AIN93 Trace Minerals, 6.8 Potassium Dihydrogen Phosphate, 1.6 Potassium Sulfate, 2.5 Potassium Citrate, 2.5 Choline Chloride (75%) and 10 AIN93 Vitamins. Animal weight was recorded weekly. On day 13 post-reserpine injection, animals were anesthetized with isoflurane, and blood samples were collected via cardiac puncture. Collected samples were processed by centrifugation to separate the serum. Serum acetate concentrations were subsequently measured using a Colorimetric Acetate Assay Kit (Cat#. ab204719; Abcam).

### Method details

#### Behavioral assays

##### von Frey test for mechanical allodynia

Mechanical allodynia was assessed by measuring hindpaw withdrawal response to von Frey filament stimulation using a SUDO up -and-down method.^67^ Rats were allowed to acclimatize for 15 min to the testing apparatus, which comprised individual clear Plexiglass boxes on an elevated wire mesh platform to facilitate access to the plantar surface of the hind paws.

##### Hargreaves test for thermal hypersensitivity

The Hargreaves apparatus was used to evaluate hypersensitivity to heat. Rats were acclimatized to the testing apparatus for 15 min before testing, which comprised individual clear Plexiglass chambers and a radiant heat source. The infrared intensity was set and cut off at 20 s. Latency expressed in seconds, was evaluated before (baseline) and at different time points after the reserpine injections.

##### Acetone test for cold-induced allodynia

Before testing, the rats were placed on an elevated wire mesh. Prior to scoring, 60 μL of acetone was applied to the plantar hind paw using a micropipette. The behaviors of the rats were then observed and scored within a 60 s interval according to the following scale: 0, no reaction; 0.5, glancing at the paw; 1, withdrawal or paw lift; 1.5, scratching, paw licking, or paw bending; 2, brisk paw withdrawal; 3, scratching over an extended period; 4, flicking; 5, extended licking of the stimulated paw.

##### Brush test for tactile hypersensitivity

To assess tactile hypersensitivity, a dynamic brush test was performed. Rats were individually placed in transparent acrylic chambers on an elevated wire mesh platform and allowed to acclimatize. A soft-bristled brush was used to apply a light stroke to the plantar surface of each hind paw, moving from heel to toe. The stimulus was repeated ten times for each paw, with a brief interval of several seconds between applications. A positive response was defined as a clear and immediate withdrawal, shaking, or licking of the stimulated paw. The total number of positive responses out of the ten trials was recorded for each animal.

##### Rotarod test for motor function

An accelerating rotarod was set so speed increased from 7 to 80 rpm over 170 s, with an acceleration speed of 2.9. Before Day 0, rats were trained on the rotarod twice daily for two days (≥2 trials per session) until performance times were stable. On the day of the experiment, three baseline trials were recorded. Latency to fall (seconds) was measured in triplicate in each trial.

#### RT-qPCR

Rats were anesthetized (5% isoflurane) and decapitated and DRG, and spinal cord were removed and collected, tissues were snap frozen in dry ice and stored at −80 °C until analysis. Tissue was homogenised using a BeadBug microtube homogenizer and BeadBug prefilled tubes with 1.5 mm Zirconium beads (Merck, cat no: Z763799). Total RNA was extracted from tissues using an RNeasy Plus mini (Qiagen, Cat# 74104) according to the manufacturer’s instructions. The concentration and purity of the isolated RNA were determined using a spectrophotometer and cDNA was synthesized from RNA using the RT2 First Strand Kit (Qiagen, Cat #330404) following the manufacturer’s instructions.

For spinal cord tissue, pre-designed RT2 qPCR Primer Assays (Qiagen, Cat. #330001) were used to quantify the expression of *Il-2*, *Il1-b*, *Il-6*, *Il-10*. For these assays RT-qPCR was performed using a standard SYBR Green-based master mix. For DRG tissue, custom-designed primers and dual labeled BHQ probes with 5′ FAM and 3′ BHQ-1 (BioSearch) were used for *Il-1α*, *Il-1β*, *Il-2*, and *Il-10* (See key resources table). For spinal cord, custom-designed BHQ-1 (BioSearch) primers for *Iba1*, *Cd68*, and *GFAP* were used (See key resources table). For these PCRs, amplification was performed using mastermix from the SensiFAST Probe Lo-ROX Kit. All RT-qPCRs were run on a Qiagen Rotor-Gene Q 5plex HRM in triplicate and normalised to the housekeeping gene *Hprt1* (Hypoxanthine-guanine phosphoribosyltransferase 1), PCR’s were cycled between 95°C for 10 s and 60°C for 30 s for at least 30 cycles. Relative gene expression was determined using the 2ˆ-ΔΔ method.

#### Shotgun metagenomic sequencing of the gut microbiome

Fecal samples were collected from each animal (reserpine-treated) after 6 weeks of diet. Samples were placed in individual tubes containing DNA stabilization buffer and then shipped to Transnetyx, Inc. (Cordova, TN, USA) for shotgun metagenomic sequencing to characterize the gut microbiome.

#### Immunofluorescence

For immunohistochemical analysis of rat tissue, spinal cord was dissected and immersion fixed in 4% paraformaldehyde in 0.1 M phosphate saline (PB) at 4°C for 24 h then washed in 0.1 M PB. Tissue was placed in 30% sucrose solution for 24 h at 4°C, and embedded in OCT and frozen sections (25 μm) were prepared using a cryostat and mounted onto Superfrost Plus slides (Fisher), dried (15 min) and stored at −20°C. Sections were blocked in PBS containing 0.3% Triton X-100 and 10% normal horse serum (NHS) (2 h, room temperature), then incubated with rabbit anti-IBA1 (1:1000, abcam, # ab178847) and Goat anti-NeuN (1:1000, Thermofisher, # PA5143586) in PBS containing 0.3% Triton X-100 and 1%NHS (overnight, 4°C). Sections were washed 4x in PBS and incubated with donkey anti-rabbit Alexa 488 (1:1000, abcam, # ab150073) and donkey anti-Goat Alexa 647 (1:1000, abcam, # ab150135) (1.5 h, room temperature). Labeled sections were imaged on Nikon C2 confocal microscope with a 40x oil objective. For each animal, z stack images were captured at 1 μm intervals through a total thickness of 10 μm. For quantitative analysis, maximum intensity projection images were generated from the Z-stacks. A semi-automated, high-throughput analysis was performed using the MicrogliaMorphology plugin,^28^ which classifies individual Iba1-positive cells into four distinct morphological categories based on 27 cellular features: resting (ramified) and activated (hypertrophic, amoeboid, and rod-like). For each image, the total number of microglia and the number of cells in each morphological category were counted. The total number of neurons was determined by counting NeuN-positive nuclei. Quantification was performed by an investigator blinded to the experimental conditions. The results were expressed as the ratio of activated microglia (sum of hypertrophic, amoeboid, and rod-like cells) to the total number of neurons, and as the percentage of resting (ramified) microglia relative to the total microglial population.

#### Preparation of spinal cord slices

Adult Sprague-Dawley rats were anesthetized with isoflurane and decapitated, and the lumbar region of the spinal cord was removed. Parasagittal slices (340 μm thick) of spinal cord were cut on a vibrotome (Leica VT1200s) in oxygenated ice-cold sucrose cutting solution that contained: 100 mM sucrose, 63 mM NaCl, 2.5 mM KCl, 1.2 mM NaH_2_PO_4_, 1.2 mM MgCl_2_, 25 mM glucose and 25 mM NaHCO_3_. Slices were transferred to a submerged chamber containing NMDG-based recovery solution for 15 min at 34 °C, equilibrated with 95% O_2_ and 5% CO_2_ and composed of: 93 mM NMDG, 2.5 mM KCl, 1.2 mM NaH_2_PO_4_, 30 mM NaHCO_3_, 20 mM HEPES, 25 mM glucose, 5 mM sodium ascorbate, 2 mM thiourea, 3 mM sodium pyruvate, 10 mM MgSO_4_ and 0.5 mM CaCl_2_, and adjusted to pH 7.4 with HCl. After the recovery incubation slices were transferred to normal oxygenated ACSF in which they were allowed to recover for 1 h at 34 °C and maintained at room temperature. Normal ACSF had the following composition: 125 mM NaCl, 2.5 mM KCl, 1.25 mM NaH_2_PO_4_, 1.2 mM MgCl_2_, 2.5 mM CaCl_2_, 25 mM glucose and 11 mM NaHCO_3_, and was equilibrated with 95% O_2_ and 5% CO_2_.

#### Whole-cell patch-clamp recordings from spinal dorsal horn neurons

Spinal cord slices were transferred to a recording chamber and continuously superfused (2 mL min^−1^) with normal artificial cerebrospinal fluid (ACSF) saturated with 95% O_2_/5% CO_2_. The temperature of the ACSF was maintained at 34 °C by an inline heater and continuously monitored by a thermistor placed in the chamber. Neurons located in laminae I and II of the superficial dorsal horn were identified using Dodt optics. Whole-cell recordings were obtained in both current- and voltage-clamp configurations using a Multiclamp 700B amplifier (Molecular Devices, USA). Patch pipettes (5–10 MΩ) were pulled from thick-walled borosilicate glass (Harvard Apparatus, UK). For the recording of spontaneous neuronal activity, current-clamp was adopted and holding at 0 pA, the pipette internal solution contained (in mM): 135 K-gluconate, 7 NaCl, 10 HEPES, 0.5 EGTA, 2 MgATP, and 0.3 NaGTP, with 1 mg mL^−1^ biocytin (pH adjusted to 7.2 with KOH, ∼290 mOsm). Offline analysis of EPSCs and IPSCs was performed using Clampfit 10 software (Molecular Devices, USA). For each neuron, a continuous, stable recording segment of at least 3 min was analyzed. Individual synaptic events were detected using a template-based search algorithm. For each recording, the event frequency (in Hz), rise time constant (Rise Tau), and decay time constant (Decay Tau) were calculated. To pharmacologically isolate and record glycine receptor-mediated inhibitory postsynaptic currents and evoked glycine receptor-mediated inhibitory postsynaptic currents (eIPSCs), a cesium-based internal solution was used, containing (in mM): 140 CsCl, 10 EGTA, 5 HEPES, 2 CaCl_2_, 2 MgATP, 0.3 NaGTP, and 5 QX-314, with 0.1% biocytin (pH adjusted to 7.4 with CsOH, ∼290 mOsm). During these recordings, the following antagonists were bath-applied to block AMPA, NMDA, and GABA-A receptor-mediated currents, respectively: 10 μM NBQX, 50 μM APV, and 10 μM gabazine. For voltage-clamp recordings of eIPSCs, neurons were held at a membrane potential of −70 mV. All electrophysiological data were digitized at 10 kHz and acquired using pClamp 10 software (Molecular Devices, USA), which was also used for offline analysis.

### Quantification and statistical analysis

All statistical analyses were carried out using GraphPad Prism software (Version 10.0, GraphPad Software, Inc.). Data are represented as mean and standard error of the mean (SEM) from independent experiments, with individual data points shown in all quantitative graphs. *p* values are indicated in the figures as follows: ∗*p* < 0.05; ∗∗*p* < 0.01; ∗∗∗*p* < 0.001. For behavioral data with repeated measures over time (mechanical withdrawal threshold, cold allodynia score; Figures 1B and 1C), a two-way repeated measures analysis of variance (ANOVA) was used, followed by Tukey’s post-hoc test for multiple comparisons. For comparisons between two independent groups, such as for serum acetate concentration (Figure 1G) and the relative abundance of individual bacterial taxa (Figures 2C and 2D), a two-tailed unpaired Student’s *t* test was performed. To compare the beta-diversity of microbiome communities, a Permutational Multivariate Analysis of Variance (PERMANOVA) was performed on a distance matrix derived from the One Codex platform. The beta-diversity distance matrix was calculated using a weighted UniFrac metric to account for both phylogenetic relatedness and relative abundance of microbial taxa. The resulting data were then visualized using a Principal Coordinates Analysis (PCoA) plot to show the clustering of samples based on their microbial community composition (Figure 2F). Statistical analysis of relative gene expression for cytokines and glial markers involving four experimental groups (Figures 4D, S1A, and S1B) was carried out using a two-way ANOVA followed by Tukey’s post-hoc test. For analysis of microglia morphology (Figures 3B and 3C), electrophysiological intrinsic properties (Figures 4E and 4F), spontaneous synaptic currents (Figures 5B, 5C, 5F, and 5G), and evoked glycinergic currents (Figure 6C), a one-way ANOVA was used, followed by Tukey’s post-hoc test for multiple comparisons.
