# Supplementary material for: Microbiome-derived metabolites alleviate chronic pain in a reserpine-induced model of fibromyalgia
Source: iScience. 2026 Mar 18;29(4):115406. doi: 10.1016/j.isci.2026.115406 (PMC13087703; doi:10.1016/j.isci.2026.115406)
Supplement: Document S1. Figures S1–S4 [file mmc1.pdf]

iScience, Volume *29*

Supplemental information

Microbiome-derived metabolites alleviate chronic pain in a reserpine-induced model of fibromyalgia

Shen Chen, Dhanya Shanmuganathan, and Wendy L. Imlach

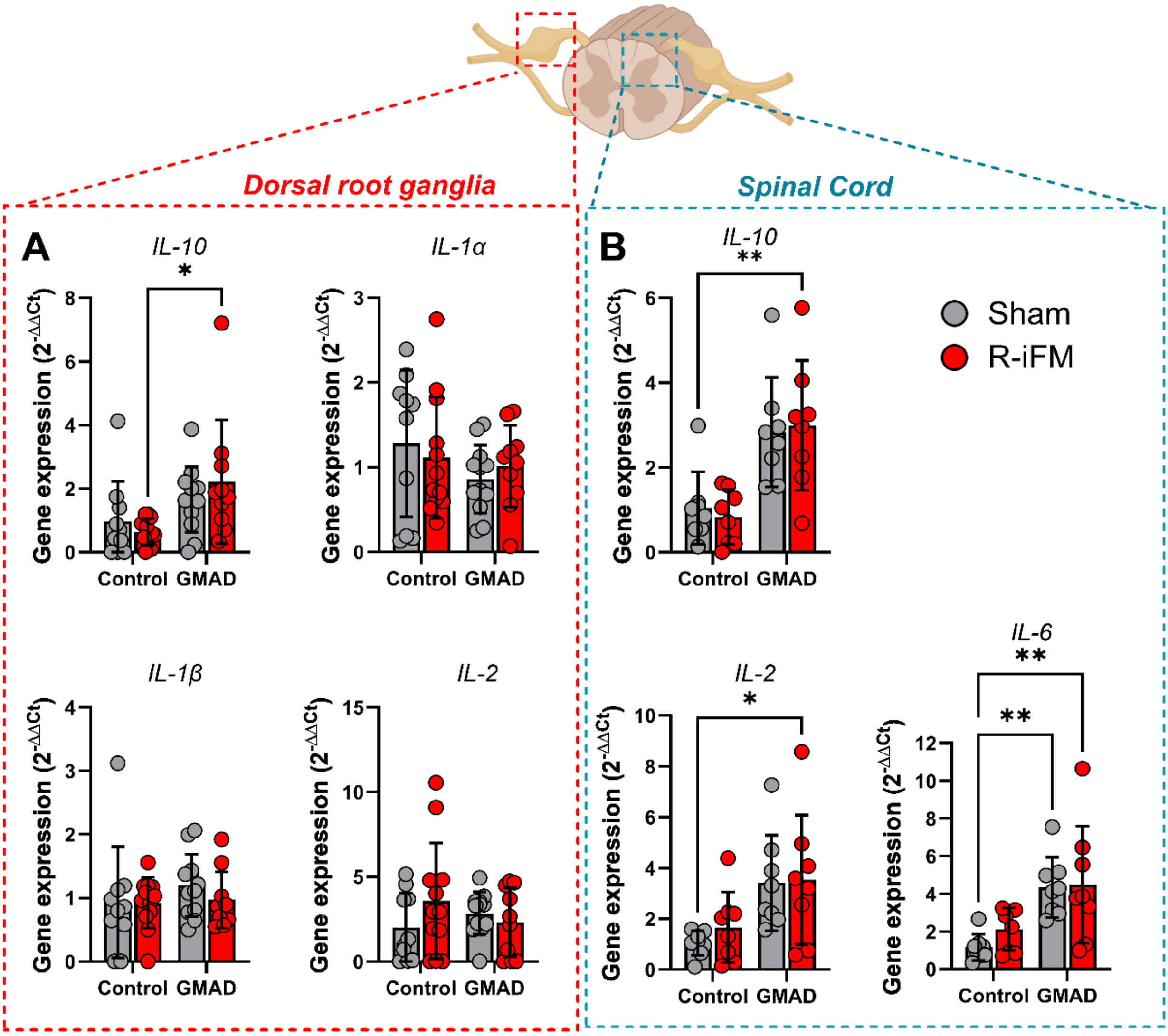

Supplementary figure 1: GMAD modulates cytokine expression in both DRG and spinal cord dorsal horn. (A) RT-qPCR analysis of cytokine expression in the DRG, shows that GMAD significantly increased the expression of the anti-inflammatory cytokine IL-10 mRNA in R-iFM rats. (B) In the lumbar spinal cord, expression of the anti-inflammatory cytokine mRNA for IL-10, IL-2 and IL-6 were elevated in GMAD groups, with significant increases in the R-iFM cohort, compared with control-diet sham group. Data are presented as fold change relative to the control sham group, mean ± SEM (n≥8 in each group with at least 3 animals in each sex). All data was normalized to the housekeeping gene HPRT1. Statistical significance was determined by two-way ANOVA followed by Turkey's post-hoc test, *p < 0.05, **p < 0.01.

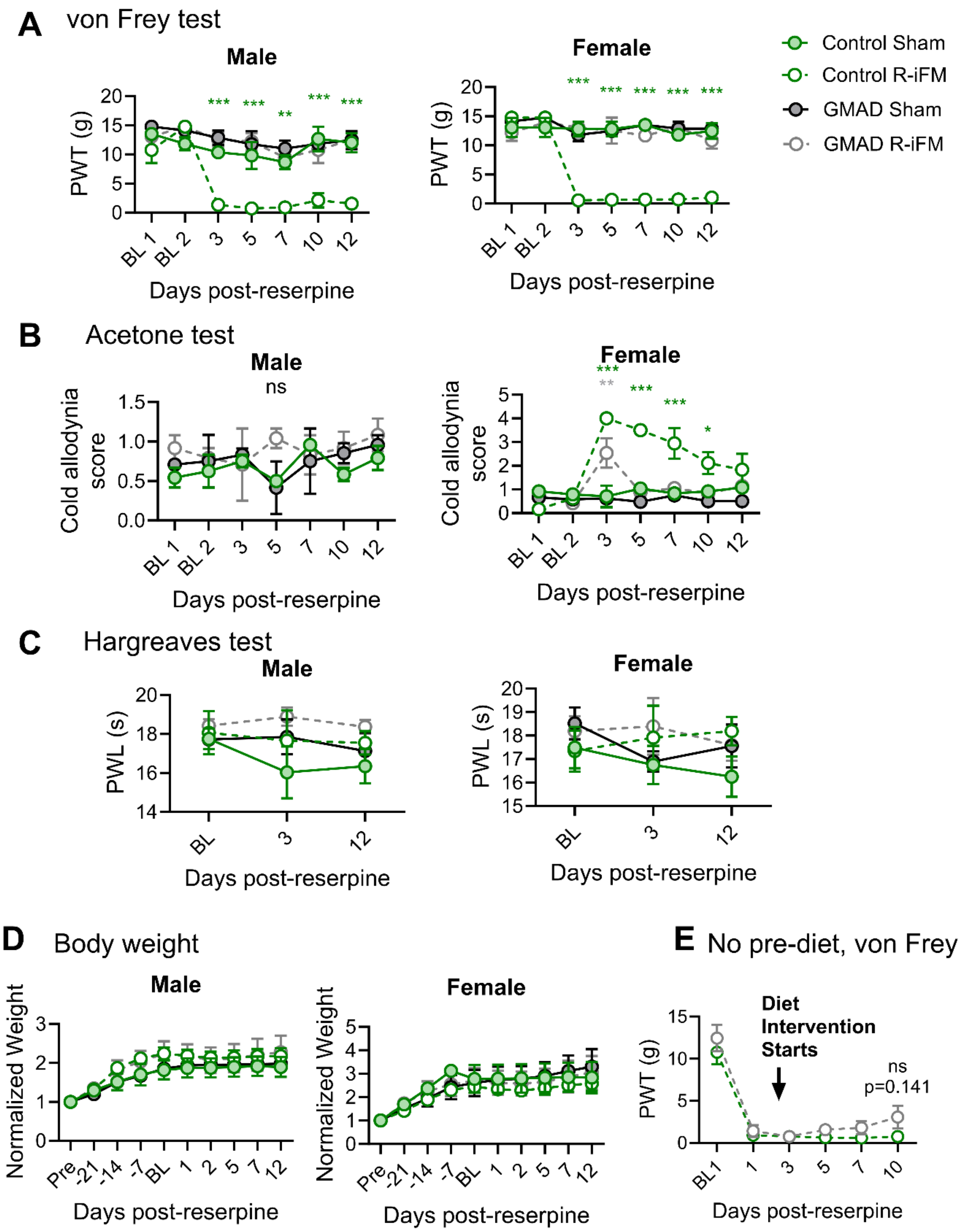

Supplementary figure 2: Behavioural tests and body weight of male and female animals and von Frey data for fibromyalgia models with no pre-diet. (A) Mechanical allodynia measured by von Frey (n > 3 rats/group, **p < 0.01, ***p < 0.001 compared to the sham group). (B) Cold allodynia in response to plantar acetone application (n > 3 rats/group, **p < 0.01, ***p < 0.001 compared to the sham group). (D) Thermal hypersensitivity (n > 3 rats/group) measured using a Hargreaves test. (D) Body weights were measured weekly prior to reserpine administration and more frequently following reserpine injections. Data are shown normalized to the initial body weight measurement for each animal. No significant differences in weight were observed between sexes, diet interventions, or experimental conditions. (E) Mechanical allodynia measured by von Frey in animals that were fed GMAD after reserpine injections started, arrow indicates the time where the diet started (n = 4 rats/group). Data are presented as mean ± SEM. PWT: Paw withdrawal threshold, PWL: Paw withdrawal latency. Statistical significance was determined by a two-way repeated ANOVA with Turkey's post-hoc test, *p < 0.05, **p < 0.01 and ***p < 0.001.

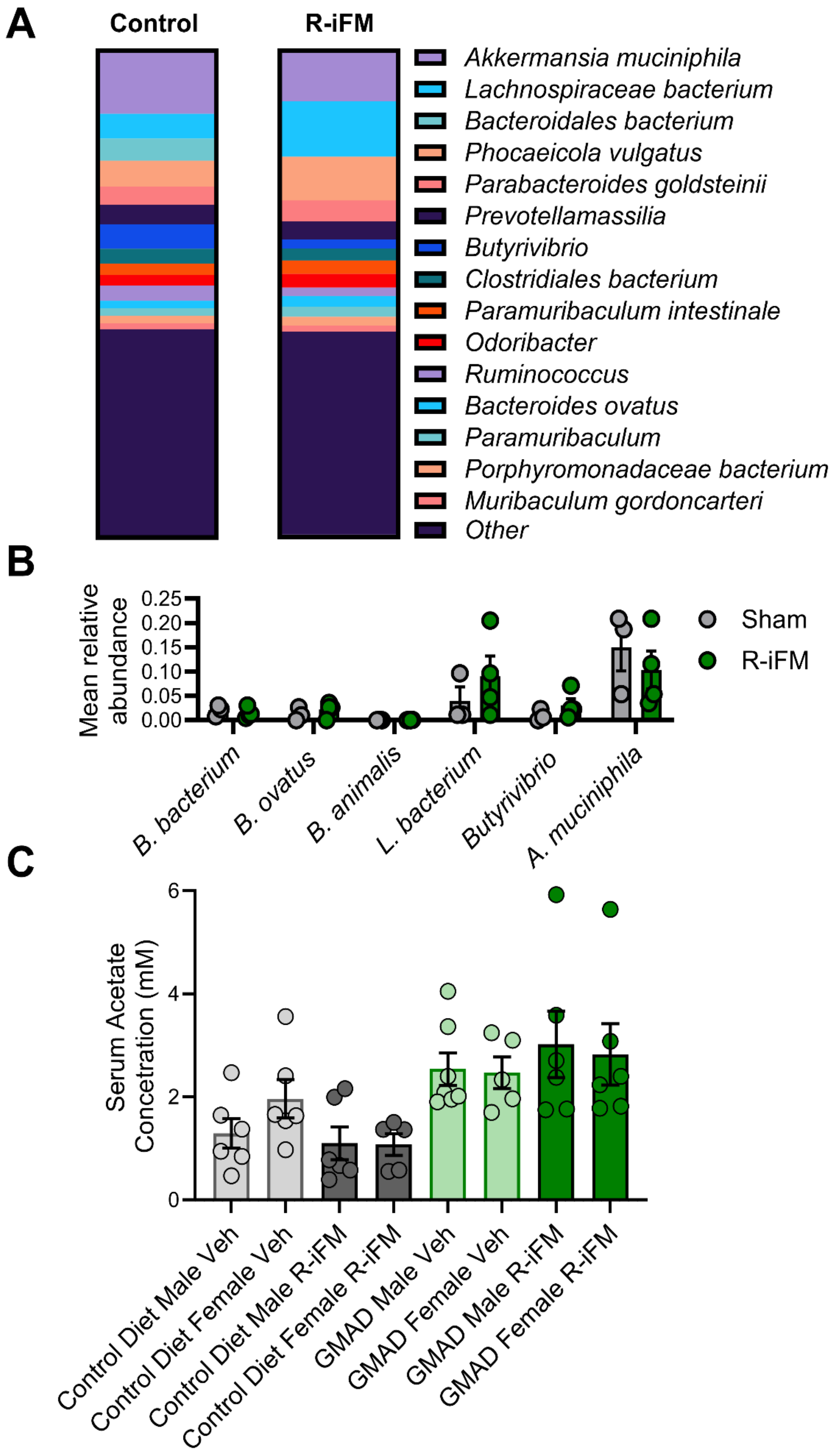

Supplementary figure 3: Reserpine does not change the bacterial composition of the gut microbiome or systemic acetate levels in control diet fed rats. (A-B) Relative abundance of bacterial genera in faecal samples, from control diet animals injected with vehicle (sham, n = 3) or reserpine (n = 4). (C) Serum acetate concentrations (in mM) for male and females within each group.

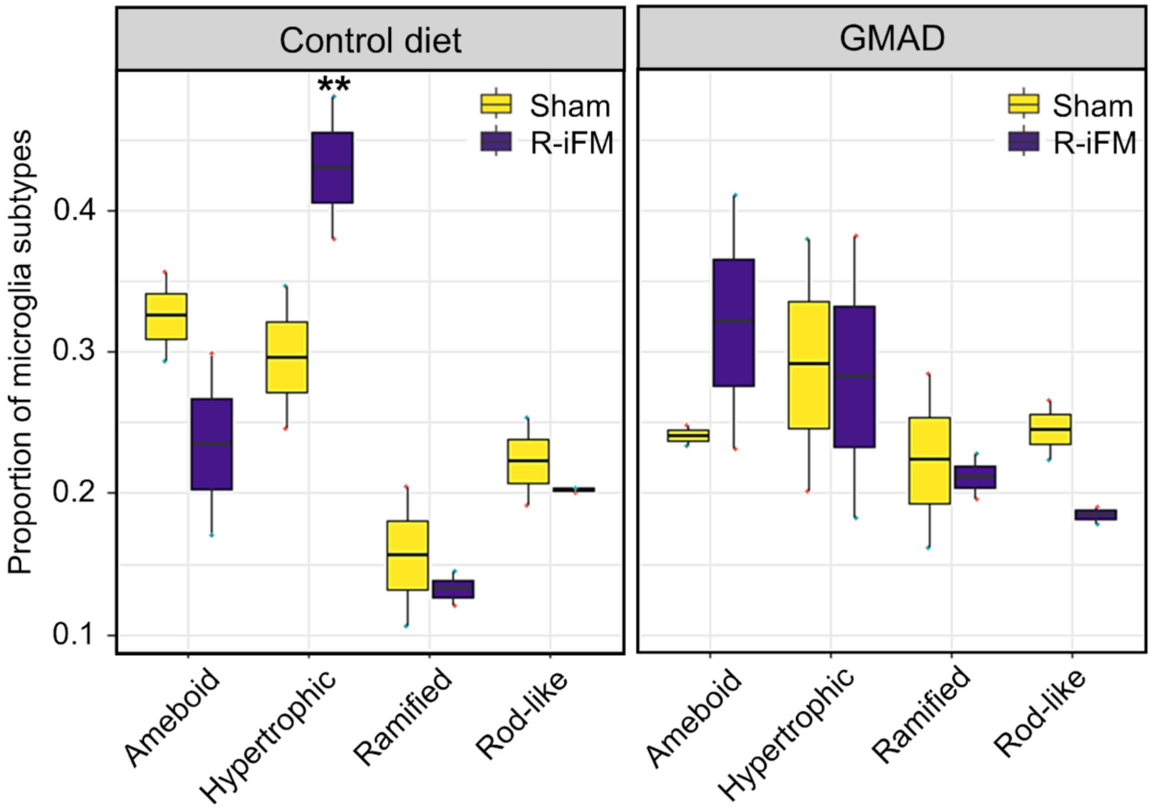

Supplementary figure 4: Microglial Morphology Clustering of both sham and R-iFM groups on the control diet and GMAD. Proportions of microglial morphological subtypes were quantified in Sham and R-iFM groups under Control or GMAD diet conditions (n > 3 rats/group, **p < 0.01, compared to sham group). Data are presented as mean ± SEM. Statistical significance was determined by a two-way ANOVA with Turkey's post-hoc test.
